# Association of Vitamin D Levels With Kidney Volume in Autosomal Dominant Polycystic Kidney Disease (ADPKD)

**DOI:** 10.3389/fmed.2019.00112

**Published:** 2019-05-24

**Authors:** Larissa Collis Vendramini, Maria Aparecida Dalboni, José Tarcísio Giffoni de Carvalho Jr., Marcelo Costa Batista, José Luiz Nishiura, Ita Pfeferman Heilberg

**Affiliations:** Nephrology Division, Universidade Federal de São Paulo, São Paulo, Brazil

**Keywords:** vitamin D, hypertension, inflammatory markers, VDR, total kidney volume (TKV)

## Abstract

Vitamin D possesses renoprotective effects beyond mineral metabolism, potentially reducing arterial blood pressure and inflammation and vitamin D enzymes (CYP24A1 and CYP27B1) as well as vitamin D receptor (VDR) contribute to its homeostasis. In the present study, we aimed to determine vitamin D association with kidney volume, blood pressure parameters and inflammatory markers in ADPKD. This cross-sectional study, conducted from August 2011 through May 2016, evaluated 25(OH)D, 1,25(OH)2D and other hormonal/biochemical serum and urinary parameters, inflammatory markers and monocyte expression of VDR, CYP24A1, CYP27B1 in 74 ADPKD patients. The height-adjusted total kidney volume (htTKV) was determined by MRI and blood pressure (BP) measured through 24-h ambulatory BP monitoring (ABPM).Vitamin D insufficiency was present in 62% of patients and CYP24A1 was overexpressed in this group, raising a hypothesis of 25(OH)D increased catabolism. Serum 25(OH)D levels and VDR expression were negatively correlated with htTKV as was VDR with IL-6, IL-10, CRP, and NFκB. A multiple linear regression analysis with htTKV as dependent variable, including hypertension, CRP, eGFR, age, time since diagnosis, VDR, and 25(OH)D adjusted for season of the year showed that only the first three parameters were independent predictors of the former. There has been no association of serum 25(OH)D and VDR expression with ABPM parameters. Present findings suggested that low levels of serum 25(OH)D and VDR expression are associated with a higher kidney volume in ADPKD patients, but do not represent independent risk factors for htTKV.

## Introduction

The increase in total kidney volume (TKV) is a prognostic biomarker of decreased renal function in Autosomal Dominant Polycystic Kidney Disease (ADPKD) (1–3). Hypertension, which occurs prior to loss of kidney function in 60% of ADPKD patients, represents a significant independent risk factor for progression of the disease, contributing to cyst expansion and intrarenal ischemia hence activating intrarenal renin-angiotensin-system (RAS) (4–6). Besides genetic factors, predictors that may lead to cyst growth and increase in TKV in ADPKD also include male gender, high salt and protein intake, caffeine consumption, level of fluid intake, gross hematuria, nephrolithiasis, proteinuria among others (7–13). Inflammation, present even in initial stages of the disease may also influence the progression of ADPKD (14). Vitamin D_3_ (cholecalciferol) is synthesized in human skin from the conversion of 7,8-dehydrocholesterol by UV radiation and then hydroxylated in the liver to give origin to the circulating form 25-hydroxyvitamin D_3_ [25(OH)D or calcidiol]. The hormonal active metabolite of vitamin D, 1,25-dihydroxyvitamin D_3_ [1,25(OH)_2_D or calcitriol], is further produced in the kidneys though the enzyme CYP27B1 (1-α hydroxylase) and functions as the ligand for the nuclear vitamin D receptor (VDR) (15). Both forms of vitamin D are catabolized by CYP24A1. Vitamin D regulates the expression of pro-inflammatory genes and might act as an anti-inflammatory hormone (16). In an experimental non-orthologous model of PKD, cholecalciferol supplementation was capable to reduce proteinuria and interstitial inflammation (17). Nevertheless, few human studies focused on the relationship of vitamin D with BP and inflammation in ADPKD (18) and its impact on predictors of disease progression remains unknown. We aimed to investigate vitamin D status, serum levels of 1,25 (OH)_2_D, the expression of regulatory enzymes [CYP24A1 and CYP27B1] and VDR and their association with BP, inflammatory markers and TKV in ADPKD patients.

## Materials and Methods

Ninety eligible participants with ADPKD confirmed by positive family history and renal cysts according to ultrasonographic diagnostic criteria by Pei et al. (19), were recruited from the outpatient Polycystic Kidney Disease Unit of the Universidade Federal de São Paulo, since August 2011 through May 2016, to participate in this study. Exclusion criteria were age <18 years old, serum calcium >10.5 mg/dL and current/past use of calcium or vitamin D. The reason for excluding users of vitamin D supplements or hypercalcemic patients relied on the necessity of vitamin D supplementation foreseen for the patients exhibiting hypovitaminosis D, as a further planned intervention study, which is still ongoing. Hypertension was defined by either measurements upon enrollment, history of hypertension or use of antihypertensive medications. Body weight, height and waist circumference were obtained and Body mass index (BMI) calculated. Body fat composition was assessed by bioelectrical impedance analysis (BIA 101 Quantum, RJL Systems, Detroit, MI). Consumption of macronutrients, vitamin D, calcium, phosphorus and caffeine were assessed through a 24-h dietary recall and daily intakes were calculated as previously described (10). After this initial clinical evaluation, enrolled patients were scheduled for one blood sample drawn following an overnight fast and a 24-h urine collection, obtained during the preceding day. Subsequently, patients were scheduled to undertake a 24-h ambulatory blood pressure monitoring (ABPM) and a magnetic resonance imaging (MRI) scan to determine total kidney volume (TKV). Patients were divided in vitamin D-sufficient (>30 ng/mL) and vitamin D-insufficient (<30 ng/mL) groups for comparisons. The study was reviewed and approved by the Ethics Advisory Committee of the Universidade Federal de São Paulo, and each patient signed the informed consent form.

### Ambulatory Blood Pressure Monitoring (ABPM)

ABPM was recorded using the automatic oscillometric monitor (Spacelabs 90207, Spacelabs Inc., Redmond, USA) with patients taking anti-hypertensive medications. Reference normal values were taken from guidelines and a reduction in BP < 10% at night-time was considered as non-dipping (20).

### Imaging Protocol

MRI was performed using a standardized respiratory-triggered, T2-weighted, axial, fat-suppressed fast-spin echo sequence without gadolinium on a 1.5-T scanner. TKV was determined from 3-mm axial T2 magnetic resonance images with renal volumetrics performed by obtaining length, width, and depth to calculate total TKV using the ellipsoid equation, with values combined from both kidneys, corrected for height (htTKV) (1).

### Clinical and Laboratory Measurements

**Figure 1:**
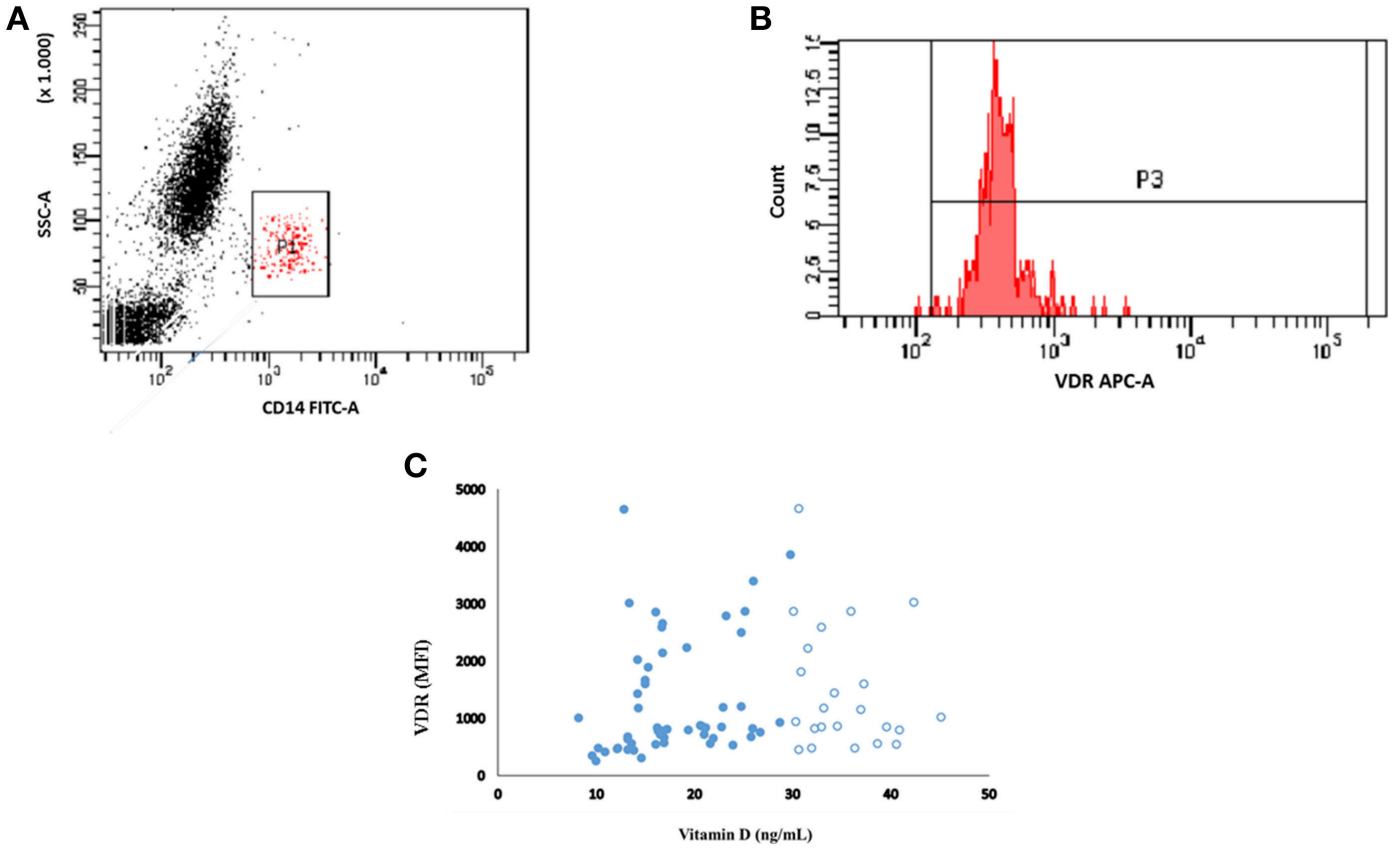
Representative flow cytometry plot showing the monocytes population (CD14+) **(A)**. Monocytes were gated into VDR **(B)**. The MFI of VDR from patients with vitamin D (25(OH)D) levels less than (closed circle) and more than (open circle) 30 ng/ml **(C)**.

Creatinine was determined by an isotope dilution mass spectrometry traceable method and estimates of glomerular filtration rate (eGFR) were obtained using the CKD-EPI equation. Stages of CKD were defined according to KDIGO. Serum calcium, phosphorus, alkaline phosphatase (colorimetric methods), urinary urea (enzymatic assay), sodium (ion selective electrode), and albuminuria (immunoturbidimetry) were measured in a Beckman Clinical Chemistry Analyzer (AU480-America Inc., Pennsylvania, USA) and intact PTH by chemiluminescence assay (Architect intact PTH, Abbott, Germany). Serum intact Fibroblast growth factor 23 (FGF-23- R&D Systems Inc., Minneapolis, MN), interleukin-6 (IL-6), interleukin-10 (IL-10), tumor necrosis factor-alpha (TNF-α), and nuclear factor kappa B (NFκB) (BD-Biosciences/eBiosciences, San Diego, CA) were determined by enzymatic immunoassays. Serum 25(OH)D was measured by chemiluminescence (Abbott Laboratories, Abbott Park, Illinois, USA) and 1,25(OH)_2_D by HPLC. Hypovitaminosis D was defined according to the K/DOQI by 25(OH)D levels <30 ng/mL. Expression of VDR, CYP24A1, and CYP27B1 in monocytes were determined by flow cytometry (BD FACSCanto, San Jose, CA), as described elsewhere (21). Figure 1 shows the monocytes characterized by CD14 and the expression of VDR as an example. Adenosine 3′:5′-cyclic monophosphate (cAMP) was determined by an immunoenzymatic kit (cAMP Biotrak enzyme immunoassay, GE Healthcare, Amersham). Sodium chloride (NaCl) intake was estimated from urine sodium and protein intake by the protein equivalent of nitrogen appearance (PNA).

### Statistical Analysis

Categorical variables were compared between vitamin D insufficient and sufficient groups using χ2 or Fisher's exact tests. Continuous variables were submitted to a normality test (Kolmogorov–Smirnov) and nonparametric tests (Mann–Whitney) were performed when appropriate. Data were expressed as mean ± SD, median and interquartile, or proportions according to the distribution of variables. Spearman's correlation coefficients measured associations between variables. A multiple linear regression analysis with htTKV as the dependent variable, including hypertension, CRP, eGFR, age, time since diagnosis, VDR and 25(OH)D adjusted for season of the year was performed. *P* < 0.05 were considered as significant and the statistical package used was SPSS (Chicago, IL, USA). Given the lack of studies investigating the association between Vitamin D status and Kidney Volume in ADPKD, the sample size calculation was based on reports aimed to find inverse correlations between 25(OH)D and IL-6 (22, 23). As aforementioned, in line with an intervention study supplementing vitamin D foreseen for the patients with hypovitaminosis D, the current sample size was estimated as 28 patients in each group with or without hypovitaminosis D, with a drop-out of 20% (*n* = 12), based on a power of 95% and significance level of 1%, calculated through GPower program, version 3.1.9.2 (Franz Faul, University of Kiel, Germany).

## Results

### Participants Characteristics

**Figure 2:**
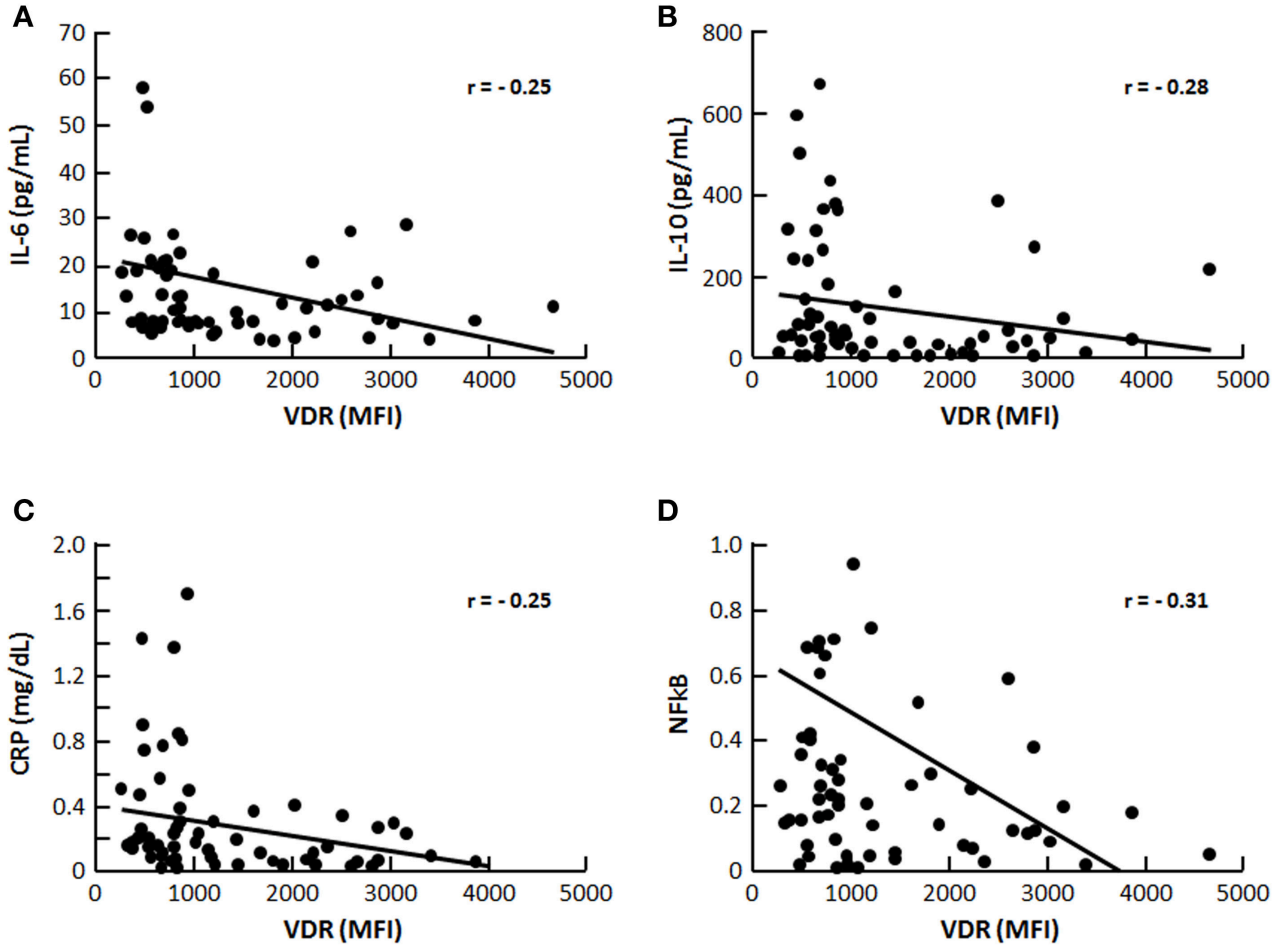
Correlation (r) between VDR and inflammatory markers **(A)** IL-6, **(B)** (IL-10), **(C)** (CRP), and **(D)** NFκB, *p* < 0.05.

**Figure 3:**
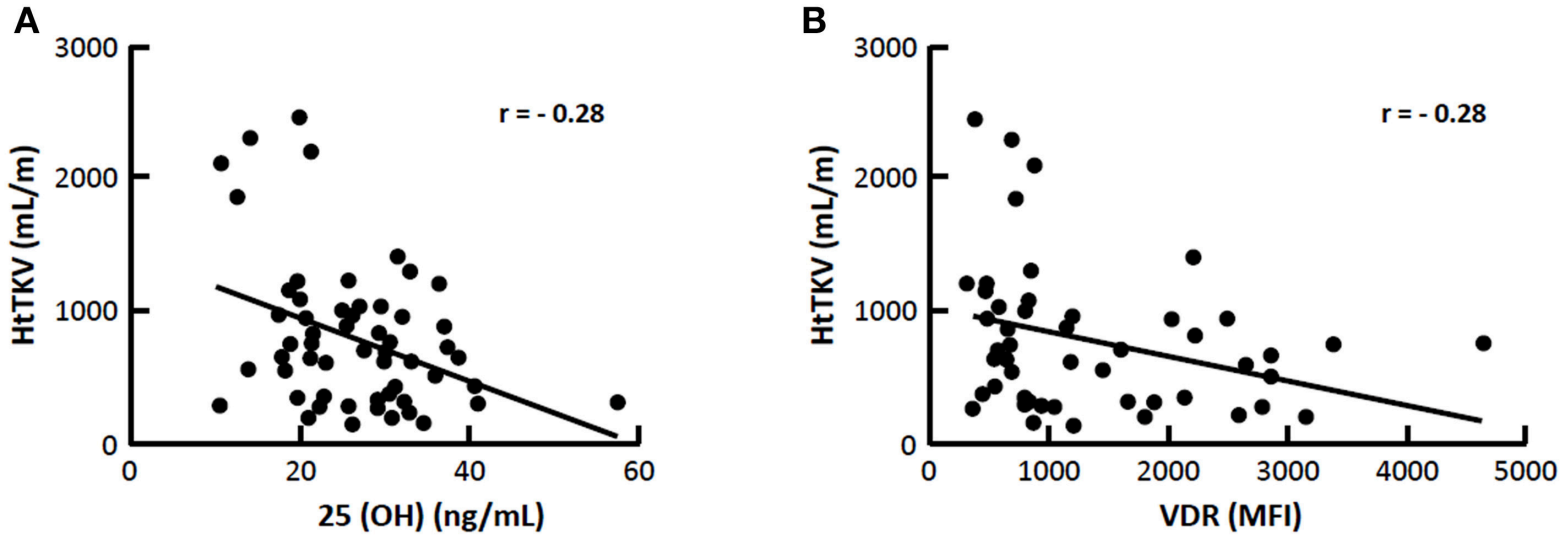
Correlation (r) between htTKV with 25(OH)D and VDR **(A)** 25(OH)D and **(B)** VDR expression, *p* < 0.05.

Of the 90 recruited patients, 14 were excluded because of previous use of vitamin D and 2 declined to participate in the study, so that 74 patients (30 M/44 F), aged 19–64 years old (40 ± 12), were enrolled. Hypovitaminosis D was observed in 46 (62%) patients, with insufficient levels (<30 ng/mL) in 39 (53%) and deficient levels (<15 ng/mL) in 7 (9%) of them. Seventy-three (73%) of patients were hypertensive and 65% were at CKD stage 1/2 and 35% at CKD 3/4. Table 1 shows characteristics of VitD-insufficient or sufficient groups, which did not differ with regard to mean age, gender, and race distribution, time since diagnosis and presence of hypertension. The percentage of patients under anti-hypertensive medication such as ACEi, ARB, or ACEi+ARB also did not differ between VitD-insufficient or sufficient groups (59 vs. 61%, *p* = 0.864; 11 vs. 7%, *p* = 0.703 and 0 vs. 7%, *p* = 0.140, respectively, data not shown). There has been no statistical difference in ABPM parameters and the percentage of non-dipping patients was similar for both groups (44 vs. 44%, data not shown). The percentage of blood collections obtained during the winter season was higher in VitD-insufficient group. Mean BMI, waist circumference and body fat were similar (Table 1), with an inadequate distribution of body fat in women (56 vs. 35%, *p* = 0.190) and in men (21 vs. 30%, *p* = 0.689), respectively for 25(OH)D <30 and >30, without statistical differences between them (data not shown). Nutritional data and median eGFR also did not differ between them. The percentage of individuals with CKD stages 1, 2, 3, and 4 was not statistically different, 37 vs. 46%; 22 vs. 28%; 30 vs. 22%; 11 vs. 4%, *p* = 0.716, respectively for VitD-insufficient vs. sufficient (data not shown in tables). Except for median 1,25(OH)_2_D and CYP24A1, that were significantly higher in the former group, mineral metabolism and inflammatory markers were not statistically different. Of the 74 patients, 10 refused to undergo MRI because of claustrophobia and 8 presented a MRI scan with incomplete coverage of both kidneys, rending it inappropriate to perform a reliable calculation of TKV. The median htTKV of vitamin D insufficient group was higher, 782 (440–10,540) mL/m (*n* = 36) when compared to vitamin D sufficient group 552 (308–817) mL/m (*n* = 20) but without statistical difference. Albuminuria and cAMP did not differ between groups. Figure 1A shows the representative plot of monocytes, Figure 1B represents the mean fluorescence intensity (MFI) of VDR gated from monocytes. There was no correlation between MFI of VDR and 25(OH)D levels (Figure 1C; Table 2). As shown in Figure 2, significant negative correlations between VDR expression with IL-6, IL-10, CRP, and NFkB were found. As depicted in Figure 3, significant negative correlations between htTKV with either 25(OH)D and VDR expression were observed. Other significant correlations, shown in Table 2, were detected between 25(OH)D and PTH, 1,25(OH)_2_D, and CYP24A1; 1,25(OH)_2_D with VDR and CYP24B1 expression and VDR with CYP27B1 expression. The multivariate linear regression analysis (Table 3) with htTKV as the dependent variable showed an independent and positive association of hypertension, CRP and negative with eGFR.

**Table 1:** Demographic, clinical, nutritional, and laboratorial characteristics of the patients.

**Parameters**	**25(OH)D (ng/mL)**			
	**Total (*n* = 74)**	**≥ 30 (*n* = 28)**	** <30 (*n* = 46)**	***p***
Age (years)	40 ± 12	39 ± 11	41 ± 12	0.493
Female/Male (*n*)	44/30	17/11	27/19	0.864
Afro-Brazilians [*n* (%)]	33 (45)	13 (46)	20 (43)	0.804
Time since diagnosis (years)	9 (4–13)	8.5 (4.5–12)	9 (4–14)	0.635
Winter season [*n* (%)]	22 (30)	2 (7)	20 (43)	<0.001
Hypertensives [*n* (%)]	54 (73)	21 (75)	33 (72)	0.759
ACEi [*n* (%)]	44 (81)	17 (61)	27 (59)	0.864
ARB [*n* (%)]	8 (15)	6 (11)	2 (7)	0.703
ACEi/ARB [*n* (%)]	2 (4)	2 (7)	0	0.140
Mean 24-h ambulatory BP (mmHg)				
Systolic	121 ± 11	121 ± 12	120 ± 11	0.971
Diastolic	77 ± 8	78 ± 8	77 ± 7	0.668
Mean daytime ambulatory BP (mmHg)				
Systolic	125 ± 12	125 ± 13	125 ± 11	0.722
Diastolic	82 ± 9	82 ± 10	82 ± 8	0.809
Mean nighttime ambulatory BP (mmHg)				
Systolic	111 ± 12	112 ± 12	110 ± 12	0.473
Diastolic	66 ± 8	68 ± 7	66 ± 8	0.266
Non-dippers [*n* (%)]				
BMI (kg/m^2^)	27 ± 5	26 ± 6	27 ± 5	0.416
Waist circumference (cm)	94 ± 14	93 ± 14	95 ± 14	0.522
Body fat (%)	27 ± 9	26 ± 8	29 ± 9	0.440
Nutritional data				
PNA (g/d)	1.1 ± 0.3	1.1 ± 0.3	1.1 ± 0.2	0.435
NaCl (g/d)	11 (7–13)	12 (6–14)	10.5 (8.5–12)	0.911
Caffeine (mg/d)	44 (4–75)	49 (21–76)	43 (4–78)	0.683
Calcium (mg/d)	566 ± 320	620 ± 288	540 ± 338	0.157
Phosphorous (mg/d)	1111 ± 506	1172 ± 442	1082 ± 542	0.203
Vitamin D (UI/d)	60 ± 73	73 ± 63	54 ± 78	0.081
eGFR (mL/min/24 h/1,73 m^2^)	76 (49–108)	88 (60–103)	72 (45–111)	0.479
Serum				
Calcium (mg/dL)	9.4 ± 0.4	9.4 ± 0.4	9.4 ± 0.4	0.654
Phosphorus (mg/dL)	3.3 ± 0.5	3.3 ± 0.6	3.2 ± 0.5	0.619
FGF-23 (pg/mL)	248 (83–2,192)	333 (85–2,277)	217 (83–1,818)	0.532
25(OH)D (ng/mL)	27 ± 9	36.5 ± 7	22 ± 5	<0.001
1,25(OH)_2_D (pg/mL)	17 (14–25)	16 (13–19)	21 (15–26)	0.018
PTH (pg/mL)	52 (33–69)	45 (33–62)	56 (36–82)	0.107
Alkaline phosphatase (UI/L)	57 (47–71)	57 (53–71)	58 (46–72)	0.688
IL-10 (pg/mL)	59 (31–202)	50 (9–243)	60 (40–148)	0.436
IL-6 (pg/mL)	9.5 (7.4–18)	11 (7–18)	8.7 (7.5–18)	0.667
TNF-α (pg/mL)	10 (8–14)	9.5 (7.8–14.5)	11 (7.8–14)	0.862
CRP (mg/dL)	0.16 (0.08–0.35)	0.17 (0.08–0.40)	0.16 (0.08–0.34)	0.696
NFκB	0.21 (0.11–0.41)	0.26 (0.11–0.41)	0.20 (0.09–0.41)	0.605
Urine				
Albuminuria [*n* (%)]	35 (47)	13 (46)	22 (48)	0.979
Urinary cyclic AMP (pMol/mL)	109 (73–237)	112 (77–228)	107 (72–251)	0.761
Expression in monocytes (MFI)				
VDR	849 (649–1,896)	1018 (792–1,896)	794 (649–1,677)	0.306
CYP24A1	548 (474–706)	483 (423–567)	640 (486–755)	0.007
CYP27B1	212 (192–250)	205 (197–221)	218 (188–285)	0.463
htTKV (mL/m)	679 (335–1,012)	552 (308–817)	782 (440–1,054)	0.124

**Table 2:** Correlation (r) between parameters.

	**25(OH)D (ng/mL)**	**1,25 (OH)_**2**_D (pg/mL)**	**VDR (MFI)**
SBP24 (mmHg)	0.02	−0.08	−0.11
DBP24(mmHg)	0.09	−0.09	−0.22
Albuminuria (μg/min)	−0.12	0.0001	−0.02
eGFR (ml/min/24 h/1.73 m2)	0.11	−0.09	0.15
Body fat (%)	−0.09	−0.08	−0.16
PTH (pg/mL)	−0.23^b^	0.04	0.04
FGF-23 (pg/mL)	0.07	0.16	0.12
1,25(OH)_2_D_3_ (pg/mL)	−0.41^a^	–	–
VDR (MFI)	0.07	0.31^b^	–
CYP24A1 (MFI)	−0.38^b^	0.07	0.17
CYP27B1 (MFI)	−0.18	0.41^c^	0.66^a^
htTKV (mL/m)	−0.28^b^	−0.07	−0.28^b^
IL-6 (pg/mL)	−0.03	−0.07	−0.25^b^
IL-10 (pg/mL)	−0.08	0.01	−0.28^b^
TNF-α (pg/mL)	−0.10	0.12	−0.05
CRP (mg/dL)	0.05	−0.22	−0.28^b^
NFκB	0.02	−0.06	−0.31^b^

a p = 0.0001;

b p < 0.05;

c *p = 0.006*.

**Table 3:** Multivariate linear regression analyses with htTKV as dependent variable.

**Independent variables**	**Multivariate analysis**		
	**Coeficiente β (EP)**	***p***	***R*^**2**^**
Hypertension	13.09 (4.40)	0.005	0.100
Age (years)	0.276 (0.13)	0.040	0.076
Time since diagnosis (years)	−0.027 (0.13)	0.842	0.001
CRP (mg/dl)	0.283 (0.106)	0.011	0.073
VDR (MFI)	−0.319 (0.155)	0.046	0.080
25(OH)D (ng/ml)	−0.2018 (0.142)	0.149	0.081
eGFR (ml/min/24 h/1.73 m^2^)	−0.446 (0.112)	< 0.001	0.346

## Discussion

Vitamin D pleiotropic effects beyond mineral metabolism, such as potential reduction of arterial BP and inflammation (24), may be of relevance inADPKD (6, 14, 25). Increased inflammatory markers in serum, urine, and fluids of cysts have been detected in ADPKD patients (14, 26, 27). Preliminary data by Gitomer et al. (28) have suggested an inverse association between serum vitamin D and kidney volume in ADPKD but to the best of our knowledge, this is the first study to evaluate the association between vitamin D, its regulatory proteins and expression of VDR with predictors of ADPKD progression.

Vitamin D insufficiency was present in 62% and CYP24A1 was overexpressed in this group. Serum 25(OH)D and VDR expression were negatively correlated with htTKV, as was VDR with inflammatory markers.

Hypovitaminosis D was more prevalent for patients who had their blood samples drawn during winter, as already evidenced in our country (29, 30). Some studies have reported an association between hypovitaminosis D with BMI (29, 31) possibly due to either the low exposure of obese individuals to sunlight or sequestration and storage of vitamin D in adipose tissue (32). We did not observe a higher BMI, waist circumference or percentage of body fat in vitD-insufficient patients, differing from previous studies by our group and others in CKD or after renal transplant (29, 31), but corroborating with Gronborg et al. (33), who found no association. Among potentially modifiable factors affecting progression of ADPKD (8), daily intakes of protein, NaCl, caffeine, calcium, phosphorous, and vitamin D did not differ between groups with or without hypovitaminosis D but both presented an intake of vitamin D under the recommended allowance (600 IU/day) and of NaCl, three-fold higher than the recommended by the American Heart Association (4 g/day). Caffeine intake was low, as already observed by our group in a previous evaluation (10).

The percentage of hypertensive patients and 24-h ABPM parameters did not differ between vitD-insufficient vs. sufficient groups diverging from epidemiological data in general population using office BP measurements (34). The employment of 24-h ABPM in the current series reinforces the reliability of our findings, who agreed with other studies not showing an association (35, 36). On the other hand, it is possible that the anti-hypertensive treatment with ARB and/or ACEi by 72% of our patients could have contributed to the lack of association between vitamin D and BP. However, even under anti-hypertensive therapy, 31/70 (44%) of patients from the current series presented a non-dipping pattern, in accordance with several investigators (19, 37, 38), who have detected it even in otherwise normotensive ADPKD subjects (38), as an early manifestation of endothelial dysfunction (39). We observed a high percentage of albuminuria (48%), particularly among hypertensive patients, corroborating with data from Chapman et al. (40), but without association with hypovitaminosis D. The use of ARB and/or ACEi might have also accounted for by the absence of such association. As cAMP accumulation plays a central role in cystogenesis (41), we determined urine levels of cAMP in the present series, but no statistical difference has been detected between vitD-insufficient or sufficient groups. The median level of eGFR and the percentage of CKD patients (stages 3/4) distributed among vitD-sufficient (26%) and insufficient groups (41%) were not statistically different (*p* = 0.716), what rendered more adequate the comparison of all parameters between groups.

Serum 25(OH)D was negatively correlated with PTH, as expected (42), although median PTH did not differ between vitD-sufficient and insufficient groups. Surprisingly, the latter presented a significantly higher median 1,25(OH)_2_D compared to the sufficient group, and a negative correlation between 25(OH)D with 1,25(OH)_2_D in the whole sample was disclosed. These unforeseen findings might have been attributed, as suggested by Need et al. (43), to a biphasic relationship between calcidiol and calcitriol depending on the level of 25(OH)D: positive whenever it is in the normal range (attributed to substrate deficiency), but negative when it is low, due to secondary hyperparathyroidism. Although inflammatory parameters were similar among vitD-insufficient and sufficient patients, an inverse association of VDR expression with IL-6, IL-10, NFκB, and CRP was observed, supporting that VDR is directly involved in the regulation of inflammatory response (16). A chronic inflammatory milieu is observed in cystic PKD kidneys, as evidenced by the large numbers of interstitial macrophages ultimately promoting cyst epithelial cell proliferation, cyst expansion, and disease progression (44). Cultured Pkd1-deficient cells express the monocyte chemoattractant protein-1 (MCP-1) and CXCL16 (C-X-C Motif Chemokine Ligand 16) and large numbers of activated macrophages surrounding the cysts have been observed in orthologous models of PKD (45). Peda et al. (44) have demonstrated that cystic epithelial cells induce renal M2-like macrophage polarization which in turn enhances the ability to promote cyst cell proliferation. IL-10 was shown to be upregulated in human ADPKD tissue and present in cyst fluid, and although this regulatory cytokine has anti-inflammatory functions, M2-phenotype requires IL-10 secretion by the macrophages and IL-10-stimulated activation of STAT3 is required for this pathological macrophage differentiation (44). M2-like macrophages have been identified in interstitial areas juxtaposed to cysts in human ADPKD kidneys, potentially promoting cyst growth by stimulating nearby cyst lining epithelial cells (46). Recently, lysine methyltranferase SMYD2 was shown to increase cystic renal cell proliferation through methylation and activation of STAT3 and the p65 subunit of NFκB (47). VDR can form a complex with the p65 subunit of NFκB to produce anti-inflammatory actions (24) and vitamin D supplementation has already been reported to help reducing circulating levels of IL-6 in other populations such as end-stage renal disease patients (21). The inverse correlation between VDR with IL-10 and NFκB observed in the present series, suggest that targeting reduction of inflammation with vitamin D or other VDR-stimulating agents may represent an effective strategy for slowing PKD progression and further studies are needed to test this hypothesis. We cannot exclude the possibility of vitamin D deficiency being a consequence rather than the cause of inflammatory response, as excess of 1,25(OH)_2_D is produced in an effort to up-regulate the VDR and 25(OH) is rapidly metabolized in this process (48). D. In the present series, median serum calcium, phosphorus, alkaline phosphatase did not differ between vitD-sufficient vs. insufficient patients. Median FGF-23 in the whole sample, composed mostly of ADPKD patients at CKD stages 1 and 2, was high if compared to CKD caused by other conditions (49, 50), being in agreement with Pavik et al. (51) who observed a four-fold increase in FGF-23 from ADPKD compared to CKD of other etiologies with similar eGFR, although no association with 25(OH) and 1,25 (OH)_2_D was detected. Accordingly, in the present sample, FGF-23 was not statistically different between groups with or without hypovitaminosis D, nor correlated with 25(OH)D or 1,25(OH)_2_D. Since both groups had been equally exposed to the use of ACEi (61 vs. 59%), the latter could have acted as a confounder, negatively regulating FGF-23 levels and disrupting the cross-talk between vitamin D and RAS (24). Moreover, as shown by Spichtig et al. (52), FGF-23 is detected in cells lining renal cysts of PKD animals but it fails to appreciably downregulate CYP27B1 due to resistance.

With regard to vitamin D regulatory enzymes, the vitD-insufficient D group presented a higher median expression of CYP24A1, which in turn correlated negatively with 25(OH)D, raising the hypothesis of vitamin D catabolism. As expected, we found a positive correlation between CYP27B1 with both 1,25(OH)_2_D and VDR. In line with the data by Spichtig et al. (52) correlations between FGF-23 and CYP24A1 have not been disclosed in the current study as well (data not shown).

Finally, the negative correlation between both 25(OH)D levels and VDR expression with htTKV disclosed in the present study, suggested a potential contributionof hypovitaminosis D to kidney enlargement in ADPKD. However, the results of the multivariate regression analysis with htTKV as the dependent variable, including hypertension, CRP, eGFR, age, time since diagnosis, VDR and 25(OH)D adjusted for season of the year showed that only the first three parameters were independent predictors of the former.

Limitations of the present study included its cross-sectional design and the need of concomitant use of anti-hypertensive therapy, which could have contributed, at least in part, to the negative results obtained with respect to blood pressure. The high proportion of hypovitaminosis D obtained in samples collected during the winter might have influenced our results. Therefore, an adjustment of the serum 25(OH)D results according to the season of the year in the multiple regression analysis was performed to take into account this confounded limitation. On the other hand, our study also has strengths such as the employment of 24-h ABPM for BP measurements.

In conclusion, present findings suggested that low levels of serum 25(OH)D and VDR expression are associated with a higher kidney volume in ADPKD patients, but hypovitaminosis D does not represent an independent risk factor for increasing kidney volume.

## Ethics Statement

The study was reviewed and approved by the Ethics Advisory Committee of the Universidade Federal de São Paulo, and each patient signed the informed consent form in accordance with the Declaration of Helsinki.

## Author Contributions

LV designed the study, performed the clinical study and the experiments, analyzed the data, and drafted the manuscript. IH designed the study, analyzed the data, provided intellectual content of critical importance to the work described, and revised the manuscript. MD, JdC, and JN participated in the analysis of data. MB helped in the conception and analysis of data.

### Conflict of Interest Statement

The authors declare that the research was conducted in the absence of any commercial or financial relationships that could be construed as a potential conflict of interest.
